# Incidental Findings of Computed Tomography Angiography in Patients Suspected to Pulmonary Embolism; a Brief Report 

**Published:** 2019-10-22

**Authors:** Mustafa Korkut, Cihan Bedel, Kürsat Erman, Serkan Yüksel

**Affiliations:** 1 Department of Emergency Medicine, Health Science University, Antalya Training and Research Hospital, Antalya, Turkey.; 2 Department of Radiology, Akdeniz University Faculty of Medicine, Antalya, Turkey.; 3 Department of Radiology, Health Science University, Antalya Training and Research Hospital, Antalya, Turkey.

**Keywords:** Pulmonary embolism, tomography, incidental findings, emergencies

## Abstract

**Introduction:**

Computed tomography pulmonary angiography (CTPA) scans are increasingly used in emergency department (ED). Therefore, the observation of incidental findings (IFs) has also increased. This study aimed to evaluate the frequency of IFs in patients who underwent CTPA.

**Methods:**

All consecutive patients that underwent CTPA scanning for pulmonary embolism (PE) rule out between January 2017 and June 2018 were analysed. Incidental findings were divided into and reported in three categories: group 1 potentially life-threatening, group 2 required follow up, and group 3 with limited clinical significance.

**Results:**

151 cases with the mean age of 61.2 ± 17.6 years were studied (54.3% female). PE was documented in 77 cases (50.9%). 448 IFs were detected (3 IFs were found per patient). 60 (13.3%) IFs were classified as group 1, 180 (40.1%) as group 2, and 208 (46.6%) as group 3. Cardiomegaly was the most frequent finding in group 1 (n=32), followed by aortic aneurysm (n=13). In group 2, pleural effusion (n=58) and pneumonia (n=36) were the most frequent incidental findings. Lung structure changes (n=92) and thoracic bone related findings (n=43) were the most common IFs observed in group 3.

**Conclusion:**

IFs were detected in the majority of patients that underwent CTPA. Most of these findings do not require follow-up or treatment. However, more than 50% of cases may require further diagnostic evaluation (40.1%) or immediate treatment (13.3%).

## Introduction

Pulmonary embolism (PE) is a challenging diagnosis in emergency department (ED) characterized by high morbidity and mortality (1). Early diagnosis and treatment can reduce the mortality rate of this disease (2). The symptoms and clinical signs of PE are non-specific and many thoracic pathologies such as pneumonia and aortic dissection cause similar symptoms (3, 4). Computed tomography pulmonary angiography (CTPA) scanning is increasingly used as a diagnostic technique in PE patients. The disadvantages of CTPA are radiation exposure, contrast induced nephropathy, and cost (5). 

The main advantage of this imaging concept is being quick and providing information on all thoracic structures including lung parenchyma (6). Another point of interest is the observation of incidental findings (IFs) on PE scanning. These findings can be irrelevant to the clinical scenario and might necessitate further investigations and treatment (7). 

Based on the above-mentioned points, the aim of this study was to evaluate the frequency of IFs in patients who underwent CTPA scanning for PE rule out.

## Methods

***Study design and setting***

This retrospective cross-sectional study was performed on patients who underwent CTPA in ED of Health Science University Antalya Training and Research Hospital, Antalya, Turkey, between January 1, 2017 and June 30, 2018. The study protocol was approved by ethics committee of the hospital (Ethics code: 2019-235). 

***Participants***

The study population consisted of all patients for whom a CTPA was ordered in the ED evaluation. The CTPA images of the patients included in the study were interpreted by two radiologist. CTPAs obtained in other hospitals, were not included if the images were not retrievable on the workstation to be submitted for interpretation.

***Data gathering***

The patients’ data were accessed via the automation system of the hospital and admission files. The CTPA was read as positive for PE if filling defects were noted in the pulmonary arterial tree. If the scan yielded a positive result for thrombosis, intravenous localization was also recorded. If there were any IFs, they were observed as well. A negative scan result was also registered. 

All patients were examined using ECLOS 16-section computed tomography scanner (Hitachi Medical Systems, Tokyo, Japan) in the ED. The CTPA images were obtained from the Picture Archival Computer System (PACS). 

IFs were classified into three categories (major, moderate, and minor) by Lumbreras et al. depending on severity (8). As in literature, in our study IFs were classified into three groups, too. Group 1 included major or potentially life-threatening findings such as abdominal aortic aneurysm and a new-found malignancy. Group 2 included moderate findings that could require diagnostic follow-up and group 3 included minor findings with limited clinical significance like anatomic variants. 

***Statistical Analysis***

Descriptive statistical analysis of all variables was performed using SPSS 18.0. Mean ± standard deviation for continuous variables and number (%) of non parametric data were calculated and reported. 

## Results

**Figure 1:**
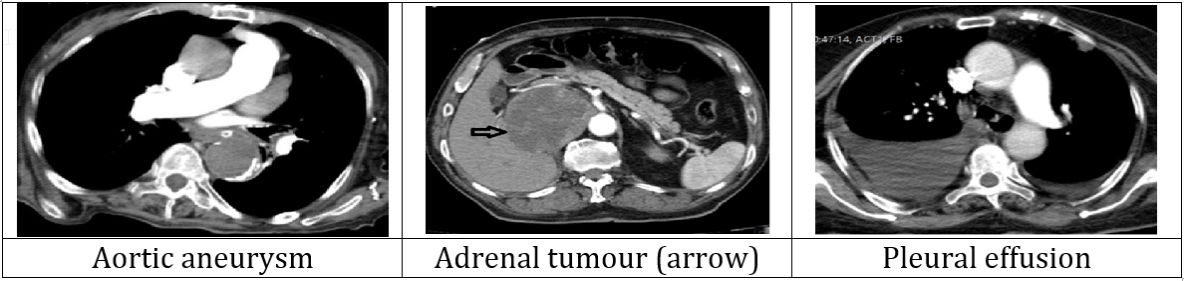
Examples of incidental finding among cases that underwent computed tomography angiography

151 cases with the mean age of 61.2 ± 17.6 years were studied (54.3% female). PE was documented in 77 cases (50.9%). Thrombus was detected in main pulmonary arteries in 27 (16.7%) patients, segmental arteries in 62 (38.5%), and subsegmental arteries in 72 (44.7%) cases. 448 IFs were detected (3 IFs were found per patient). 60 (13.3%) IFs were classified as group 1, 180 (40.1%) as group 2, and 208 (46.6%) as group 3 (table 1). Cardiomegaly was the most frequent finding in group 1 (n=32), followed by aortic aneurysm (n=13) and malignancies such as adrenal tumor. In group 2, pleural effusion (n=58) and pneumonia (n=36) were the most frequent incidental findings. Lung structure changes (n=92) and thoracic bone-related findings (n=43) were the most common ones observed in group 3. Figure 1 shows examples of some detected IFs.

## Discussion

The present study showed that CTPA detected IFs in the majority of patients. Most of these findings do not require further evaluation or treatment. However, some may require follow-up and immediate treatment. In this study, 13.3% of detected IFs required emergent evaluation and 40.1% were in need for further diagnostic evaluation.

 CTPA has become the imaging test of choice in many institutions for diagnosis of PE because of high interobserver agreement, detection of other pathologies and cost-effectiveness (9). When we compare other imaging modalities, such as ventilation / perfusion scintigraphy, with CTPA, one of the most important advantages of CTPA is allowing us to perform pulmonary vascular examination as well as parenchymal, mediastinal, cardiac, pleural structures, thoracic wall, and upper abdominal organ evaluations (9, 10). Another interesting point is the incidence of IFs in many patients, even though embolism is detected in approximately 1 in 5 with this imaging method. Although most of these are benign, age-related changes, there may be findings that may require immediate treatment and follow-up. 

In this study, the prevalence of PE in CTPA was 51%. In previous studies this rate ranged from 19% to 79% (11). 

In this study, IFs were very common (about 3 findings for each patient). But more importantly, almost half of the patients had findings that required follow-up. 

**Table 1:** Incidental findings in cases suspected to pulmonary embolism who underwent computed tomography angiogarpgy

**Incidental findings **	**Number (%)**
**Group1 (n = 60)**	
Bronchial cancer	3 (5.0)
Aortic aneurysm	13 (21.7)
Liver metastasis	5 (8.3)
Adrenal tumour	1 (1.7)
Pulmonary mass	2 (3.3)
Mediastinal mass	4 (6.7)
Cardiomegaly	32 (53.3)
**Group 2 (n = 180)**	
Pulmonary nodules	21 (11.7)
Pleural effusion	58 (32.2)
Cholecystolithiasis	4 (2.2)
Pneumonia	36 (20)
Thyroid nodule	13 (7.2)
Pancreatitis	1 (0.6)
Pericardial effusion	6 (3.3)
Significant atelectasis	17 (9.4)
Hiatal hernia	8 (4.4)
Ascites	2 (1.1)
Ileus	1 (0.6)
Pulmonary artery enlargement	10 (5.6)
Atrophic kidney	1 (0.6)
Bochdalek hernia	2 (1.1)
**Group 3 (n = 208)**	
Renal cyst	23 (11.1)
Hepatic cyst	4 (1.9)
Lung structure changes	92 (44.2)
Splenomegaly	3 (1.4)
Adenopathy	10 (4.8)
Coronary artery calcification	31(14.9)
Bone finding	43 (20.7)
Hemangioma	1 (0.5)
Atrophic kidney	1 (0.5)

Group 1: need for emergent treatment.

Group 2: need for further diagnostic evaluation.

Group3 : minor findings with no need for further evaluation.

Approximately 13.3% of the studied cases had findings requiring immediate treatment; mostly pleural effusion (n=58). This frequency is consistent with a previous study on a similar population (12). Frequency of patients with group 1 category IFs varied between 15% to 24% in previous studies (13, 14). Hall et al. evaluated 589 patients and found a greater frequeny of IFs in need of emergent evaluation compared to our study. However, their high rate is due to inclusion of patients with major or potentially life-threatening findings and those with moderate findings that required diagnostic follow-up in the same category. 

The prevalence rate of pneumonia requiring follow-up has been reported to be 7.1% in CTPAs in one study (14). In our case, the prevalence was (approximately 8%); however, it is significantly lower than that observed by Hall et al. (13). 

The prevalence of lung nodules requiring follow-up was reported to be 25% in those undergoing CTPA (13). In our study, this prevalence was significantly lower. This can be partly due to the number of sections and image quality. Since the number of CTPA slices in our study was low, incidental findings such as lung nodules could be expected to be less. 

We also observed a high rate of minor findings with no need for further examination. This trend was also observed in other studies examining the prevalence of incidental findings in CTPAs (13, 15). Turkvatan et al. reported a 56.2% incidence for clinically insignificant findings in 242 cohorts of patients screened with 16-line CT (16). However, Perelas et al. reported 48.1% minor findings with no need for further examination, without classifing them by severity, in a cohort of 641 patients also scanned using a 16-slice CT (14). 

More importantly, relatively old studies reported innocent findings with diagnostic value for alternative diagnosis (9, 17). We believe that this ratio will increase with increase in image quality. 

It seems that, emergency physicians should be aware of these important IFs, and EDs should also develop mechanisms to ensure appropriate follow-up for these patients.

## Limitation

The study was conducted in a single center and in a retrospective manner and these results may not apply to different patient populations. Lack of patient follow-up is one of our most important limitations. In some cases although the findings may appear to be characteristic tomography findings, the final histopathological diagnosis may be different. This may have caused overreporting of some findings, such as malignancy.

## Conclusion

The present study showed that CTPA detected IFs in the majority of patients. Most of these findings do not require follow-up or treatment. However, some may require follow-up and immediate treatment. In our study, 13.3% of IFs required emergent evaluation and 40.1% were in need for further evaluation according to their symptoms. 
